# Efficient Wastewater Treatment and Removal of Bisphenol A and Diclofenac in Mesocosm Flow Constructed Wetlands Using Granulated Cork as Emerged Substrate

**DOI:** 10.3390/toxics11010081

**Published:** 2023-01-15

**Authors:** Salma Bessadok, Khadija Kraiem, Fatma Arous, Karim Suhail Al Souki, Dorra Tabassi, Safa El Toumi, Atef Jaouani

**Affiliations:** 1 Bioresources, Environment and Biotechnology Laboratory (LR22ES04), Higher Institute of Applied Biological Sciences of Tunis, University of Tunis El Manar, Tunis 1006, Tunisia; 2 Department of Environmental Chemistry and Technology, Faculty of Environment, Jan Evangelista Purkyně University in Ústí nad Labem, Pasteurova 3632/15, 400 96 Ústí nad Labem, Czech Republic

**Keywords:** wastewater treatment, constructed wetlands, endocrine-disrupting compounds, emerged substrates, granulated cork

## Abstract

Constructed wetlands (CWs) are considered as low-cost and energy-efficient wastewater treatment systems. Media selection is one of the essential technical keys for their implementation. The purpose of this work was essentially to evaluate the removal efficiency of organic pollution and nitrogen from municipal wastewater (MWW) using different selected media (gravel/gravel amended with granulated cork) in mesocosm horizontal flow constructed wetlands (HFCWs). The results showed that the highest chemical oxygen demand (COD) and ammonium nitrogen removal of 80.53% and 42%, respectively, were recorded in the units filled with gravel amended with cork. The influence of macrophytes (*Phragmites australis* and *Typha angustifolia*) was studied and both species showed steeper efficiencies. The system was operated under different hydraulic retention times (HRTs) i.e., 6 h, 24 h, 30 h, and 48 h. The obtained results revealed that the COD removal efficiency was significantly enhanced by up to 38% counter to the ammonium rates when HRT was increased from 6 h to 48 h. Moreover, the removal efficiency of two endocrine-disrupting compounds (EDCs) namely, bisphenol A (BPA) and diclofenac (DCF) was investigated in two selected HFCWs, at 48 h HRT. The achieved results proved the high capacity of cork for BPA and DCF removal with the removal rates of 90.95% and 89.66%, respectively. The results confirmed the role of these engineered systems, especially for EDC removal, which should be further explored.

## 1. Introduction

Water pollution has always been among the foremost environmental concerns since it directly affects human health. The World Health Organization (WHO) has declared the existence of inadequate sanitation systems in many parts of the world, mainly in several low- and middle-income countries [1]. In addition, wastewater treatment plant (WWTP) implementation and maintenance are costly worldwide and are usually challenging due to the footprint, facilities, energy consumption, personnel skills, and competences required [2,3]. In this sense, constructed wetland (CW) technologies are considered as an effective tool and cost-effective nature-based solution that can effectively improve the water quality and pollution treatment [4,5]. These systems possess a great potential for application in developing countries, mainly in rural areas, as they are designed to operate with low investment and maintenance costs with an ultimate fate in agricultural reuse [6,7]. Wastewater contains a large amount of inorganic and organic pollutants. Nevertheless, endocrine-disrupting compounds (EDCs) have recently raised scientific attention, revealing serious environmental issues worldwide [8]. These micropollutants caused a consequential environmental pollution affecting wildlife and public health due to their hormone-like behaviors and critical carcinogenic properties [9,10]. More particularly, bisphenol A (BPA) and diclofenac (DCF) have been widely detected in municipal secondary effluents [11,12,13]. According to the U.S. Environmental Protection Agency (USEPA), more than one million pounds of BPA are released annually in the environment [14] through industrial and municipal wastewaters contaminating surface water (0.001–92 mg/m^3^), groundwater (0.001–20 mg/m^3^), and even more potable water [15,16]. Furthermore, DCF, a well-known anti-inflammatory drug (NSAIDs), is one of the pharmaceuticals included in the commission implementing decision (EU) on March 2015 watch list as it is highly detected in water matrices [17,18,19]. These micropollutants, if discharged directly without treatment, cause a potential safety hazard to water sources, thus deepening the crisis of freshwater resources [20,21]. Considering the various harmful effects of EDCs, wastewater remediation systems involve various treatment methods such as biological, physical, and chemical treatments. Some of the reported methods for EDC removal from water include adsorption [22,23], nanomaterials [24], ozonation [25], biodegradation [26], and advanced oxidation processes (AOPs) [27,28]. However, the biological persistence and physico-chemical characteristics of these compounds have presented a few limitations for these technologies as the degradation process fails to eliminate the non-organic EDC molecules and can potentially form different by-product residuals. CWs appear as one of the most promising eco-tech treatment methods that encompass different removal processes (biological, chemical, and physical) [29,30]. The design of the CWs in either vertical flow (VFCW) or horizontal (HFCW) could play a major role in wastewater treatment. VFCWs mostly rank next to HFCWs in performance, except for the total nitrogen removal [31]. Studies evaluating and comparing different types of the efficiency of CW systems on the removal of EDCs are still scarce. Nonetheless, HFCW systems are a better option for EDC removal because the wastewater passage in the system activates several mechanisms that promote their removal without the need for effluent recirculation or system maintenance [32]. For example, studies about BPA removal have achieved approximately 70–90% rates in pilot-scale HFCW systems based on two parallel units. This system, when followed by a larger unit, presented an overall removal of 85–99% [33]. DCF was classified as a fast-photodegradable compound that could be removed under both anaerobic and aerobic conditions [33]. Ilyas [34] studied the DCF removal efficiency in both HFCWs and VFCWs and the removal rates were around 56 ± 32% and 50 ± 17%, respectively. Substrate media selection is one of the essential technical keys for the achievable process of CWs as it controls the environmental conditions inside the porous spaces. Nevertheless, systems based on conventional substrates such as gravel and sand may be confronted with several problems that negatively affect the removal performance [35]. These issues present a real challenge to traditional substrates and inspire the investigation and development of alternative emerged substrates in CWs [36]. Recent studies have reported the use of biochar and light expanded clay aggregates (LECA) as emerged substrates with high removal efficiencies [37,38]. However, these materials require high energy consumption for pyrolysis and manufacturing, and thus cannot be used in large-scale environmental remediation due to their excessive cost and poor stability [39]. On the other hand, cork presents an immense potential to be valued and applied in wastewater treatment considering its chemical composition, cellular structure, and high adsorption capacity. The world area of cork oak forests is about 2,306,000 ha, of which 37.5% is in North Africa and 90,423 ha in Tunisia, which makes granulated cork a low-cost locally available material [40]. It is noteworthy to mention that few studies have shed light on the remarkable characteristics of cork, and therefore data about the mode of action in CW technologies is still scarce. Within this context, the current work aims to evaluate the wastewater treatment in mesocosm-scale HFCWs and two representative EDCs removal (i.e., BPA, DCF) with an emphasis on the effect of granulated cork as an emerged substrate.

## 2. Materials and Methods

### 2.1. Influent Characteristics Used in This Study

The constructed wetland (CW) system was fed with urban domestic wastewater arriving at EL MENZAH Wastewater Treatment Plant (WWTP) with GPS coordinates: 36°49′59″N, 10°10′55″E. Table 1 shows the main quality characteristics of the influent wastewater compared to the reuse standards. To assess the removal of endocrine-disrupting compounds (EDCs), bisphenol A (BPA) and diclofenac (DCF) were spiked into the wastewater at a concentration of 20 µg L^−1^ to ensure their detection [41].

### 2.2. Mesocosm-Scale Constructed Wetlands Design

Six laboratory-scale HFCWs (i.e., H1FCW, H2FCW, H3FCW, H4FCW, H5FCW, H6FCW) with the following dimensions (59 cm length × 39 cm width × 30 cm height), were designed and constructed from polyethylene plastic and installed at EL MENZAH Wastewater Treatment Plant (WWTP). Each HFCW system was evaluated in triplicate (Table 2). In order to select the most appropriate substrate and plant species, the units were filled with two types of substrate media (gravel and gravel amended with granulated cork) and were planted with two species of macrophytes (*Phragmites australis* and *Typha angustifolia*), mostly abundant in the valleys of Tunisia and commonly used in CW implementation as they reduce wind speed, support sedimentation, and supply carbon for denitrification [45] using six stems per wetland as follows: two units were planted with six plants of *Phragmites australis*, two units with *Typha angustifolia*, and two unplanted used as the control units. Details of the different configuration modes are reported in Table 2.

### 2.3. Experimental Scheme and Operation Mode

**Figure 1:**
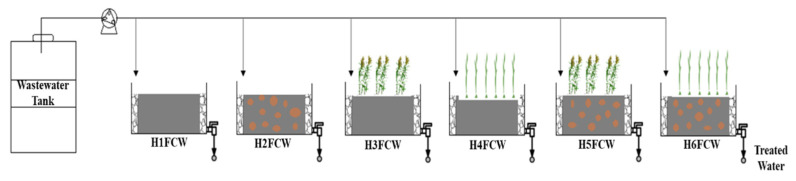
Experimental scheme of the mesocosm horizontal flow constructed wetlands (HFCWs).

A schematic diagram of the laboratory scale of HFCWs is shown in Figure 1. The different units of HFCWs (0.49 m) were filled with fine gravel (5–10 mm grain size) or a mixture of fine gravel and granulated cork with grain sizes of 2.83 to 5 mm, in the treatment zone, whereas 0.05 m in the inlet/outlet zones were filled with coarse gravel (25–40 mm grain size). The inlet and outlet compartment design ensured water level control and easy sampling. The inlet arrangement was comprised of a polyurethane pipe with a 10-mm diameter perforation, placed just above the substrate. The outlet arrangement was comprised of a 32-mm diameter perforated PVC pipe. The down applied saturation zone was maintained by a siphon structure at the outlet. 

The experiments were carried out over a period of five months (150 days). Through the adaptation period, the wetlands were fed with water for up to 30 days (50% of raw wastewater and 50% of tap water) to establish the growth of plants and biofilm in the media. After the adaptation period, the experimental systems were operated with 100% raw wastewater by batch feeding mode at a hydraulic retention time (HRT) of 48 h for over 60 days. For experimental optimization, each HRT (i.e., 30 h, 24 h, 6 h) was also tested in all configurations for 20 days. 

### 2.4. Chemicals and Standards

All chemicals and reagents used were of analytical reagent grade. Diclofenac sodium salt and bisphenol A of high purity grade (>99%) were purchased from Sigma-Aldrich (Saint-Louis, MO, USA). Stock solutions of individual compounds were prepared in methanol at 1 mg L^−1^ and kept at −18 °C. The silylation derivatization reagent N,O-bis [trimethylsilyltrifluoroacetamide] with 1% trimethylchlorosilane (BSTFA 1% TMCS) was obtained from Restek (Bellefonte, PA, USA). Supel-Select HLB SPE cartridges (200 mg/6 mL) and 0.45 μm Whatman glass microfiber filters were purchased from Sigma-Aldrich (Saint-Louis, MO, USA). HPLC grade ethyl acetate, methanol, and acetonitrile were purchased from Sigma-Aldrich (Saint-Louis, MO, USA). Ultrapure water (Milli-Q) was produced by a Millipore apparatus (18.2 MΩ cm^−1^ resistivity).

### 2.5. Sampling and Conventional Parameters Analysis

Physico-chemical analyses were conducted weekly for the influent and effluent of the different HFCWs. Chemical oxygen demand (COD), NH_4_-N, and NO_3_-N were measured with Hach Lange test cells (LCK 400, 304, and 349, respectively, Düsseldorf, Germany) on a spectrophotometer (DR 1900, Hach Lange/Dortmund, Germany). Electrolytic conductivity (EC) and pH were determined by using a multi-parameter water quality meter (SensoDirect 150, Loviband/Dortmund, Germany). 

### 2.6. Endocrine-Disrupting Compounds (EDCs) Analysis

BPA and DCF analyses were performed weekly using pre-cleaned amber bottles. To separate the dissolved phase from the suspended solid matter (SSM), the water samples were directly filtered using 0.45 μm Whatman glass microfiber filters. Targeted EDC residues in filtered water were extracted using solid phase extraction (SPE).

#### 2.6.1. Solid Phase Extraction (SPE) 

The analytical method, based on SPE extraction, was performed according to Ben Sghaier [46] for the extraction/pre-concentration of EDCs in filtered water samples (500 mL) using hydrophilic–lipophilic-balanced (HLB) copolymer SPE cartridges. Briefly, SPE cartridges were placed on a Vac Elut SPS 24 Manifold from Agilent Technologies and conditioned sequentially with 3 mL of ethyl acetate/methanol (1/1, *v/v*), 3 mL of methanol, and 3 mL of ultrapure Millipore-Q water (pH = 2). The extraction of filtered water was achieved at a flow rate of ~5 mL/min. Then, cartridges were washed with 3 mL of methanol–water (2/3, *v/v*) and dried under vacuum for 1 h. Elution was proceeded with 9 mL of ethyl acetate/acetone (1/1, *v/v*) at a flow rate of 1–2 mL/min. Extracts were evaporated until dryness under a gentle stream of nitrogen and transferred into the GC injection vial by solubilizing in 50 µL of acetonitrile. Finally, the derivatization reaction was performed at 65 °C for two hours by adding 50 μL of BSTFA + 1% TMCS and vortex-mixed for 1 min. The derivatives were kept to room temperature for 15 min, prior to GC-MS analysis.

#### 2.6.2. Chromatographic Analysis

The derivatized samples were analyzed in a GC-MS Shimadzu, model TQ8040 (Japan) equipped with an automatic sampler Shimadzu AOC-20S (Japan). All injections in the GC were performed in a volume of 1.0 μL. The injector was operated at 280 °C in splitless mode and the chromatographic column was a Rxi-5MS (fused Silica) from Restek (Bellefonte, USA), with 30 m long, 0.25 mm thick, and internally coated with a 0.25 μm thick film. Carrier gas was helium with 99.999% purity with a flow rate of 1.0 mL/min. The GC oven program started at 100 °C and was maintained for 2 min, then the temperature was incremented at 5 °C per minute to reach 250 °C, then immediately incremented at 3 °C per minute until it reached 300 °C where it was held for 2.33 min. Each targeted compound was identified based on the retention time (RT) and the mass spectrum (*m/z*) from the chromatogram of the standard solutions acquired in full scan (FS) mode. Finally, the quantitative analyses were conducted in selected ion monitoring mode (SIM). Peak integrations were performed on the SIM chromatogram using Labsolutions software. The chemical formula/structure, molecular weight, molecular mass of the derivative compounds, quantifier ions (*m/z*), and correlation coefficient (R^2^) are given in Table 3. 

The endocrine disrupting compound (EDC) removal efficiencies were estimated as the percentage change in concentration after treatment using the following equation:

$$\mathrm{EDC}\;\mathrm{removal}\;\mathrm{efficiency}=\frac{\mathrm{Ci}-\mathrm{Ce}}{\mathrm{Ci}}\times 100\;\%$$

where Ci = EDC concentration in the influent and Ce = EDC concentration in the effluent.

### 2.7. Microscopic Analysis of the Emerged Substrate

The granulated cork morphology was examined using a scanning electron microscope (Thermoscientific Q 250) coupled with energy dispersive X-ray (SEM/EDX). The 3D images of the observed surfaces are illustrated in the results. 

### 2.8. Statistical Analysis

Statistical testing was performed using the STATISTICA 3 software (http://www.statsoft.com, accessed on 10 September 2022) based on analysis of variance (ANOVA). Means were compared by the Fishers least significant difference (LSD) test and differences were considered statistically significant when *p* < 0.05. Three replicates per each pilot were considered.

## 3. Results and Discussion

### 3.1. Cork Characterization

**Figure 2:**
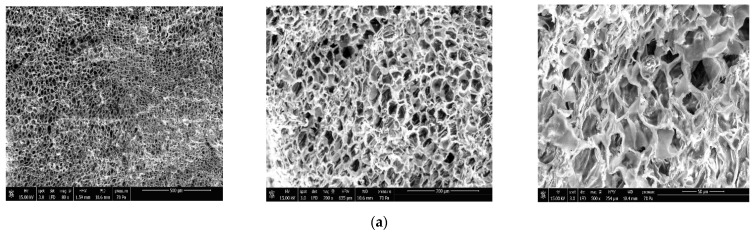

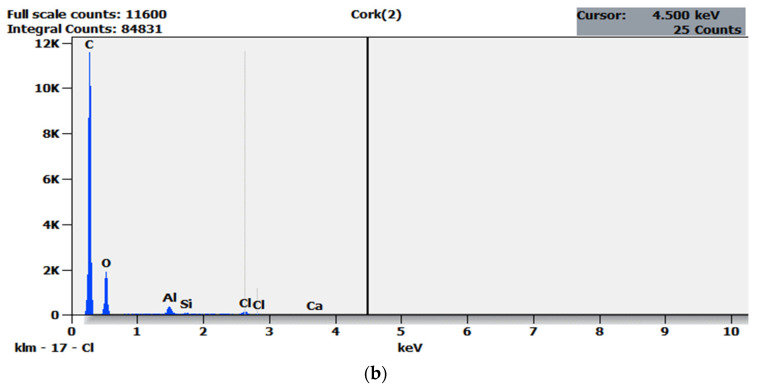
(**a**) Scanning electron microscopy (SEM) images and different magnifications of natural cork and (**b**) elemental analysis (EDX) at different magnifications of natural cork.

To better understand the cork’s morphological characteristics and their effect on wastewater treatment, scanning electron microscope (SEM) and elemental analysis (EDX) were conducted. Based on the SEM micrographs presented in Figure 2a, the cork showed a unique microscopic feature compared to other lignocellulosic biomaterials with hollow prismatic cells, presenting macropores with a diameter of about 30–40 μm (>50 nm according to IUPAC). Additionally, no internal porosity was apparent. Dordio [47] showed that the apparent porosity was fairly large through the inter-granule void space and the porous cellular surface, which can absorb and remove considerable amounts of water. Pereira [48] described the cork tissue as a compact foam with a regular honeycomb arrangement that lacked intercellular voids. Regarding the cell type, this biological tissue is homogeneous, with the cell rows parallel to each other with prism bases aligned in staggered positions.

In terms of the elemental analysis, the EDX spectra depicted in Figure 2b showed the predominance of carbon as the major component of granulated cork, representing different hydrophobic biopolymers (suberin, lignin, and other extractives). Suberin is a polymeric macromolecule of aliphatic nature that contains two types of monomers, glycerol and long chain fatty acids and alcohols. These monomers present the structural component, and its removal destroys the cell membrane integrity [48]. Unlike suberin, lignin is not specific to cork, presenting the second most important structural cell component. On the other hand, oxygen is the counterpart of carbon in the aliphatic chains of suberin and aromatic rings of lignin. Impurities of Al Si, S, Cl, and Ca were also detected. These results agree with what has been reported in previous studies by Dordio [47] and Pirozzi [49], which revealed the cork potential based on its unique characteristics, allowing for “green”, sustainable, and efficient procedures that are currently pertinent to the environmental concerns.

### 3.2. Chemical Oxygen Demand (COD) Removal Efficiency

**Figure 3:**
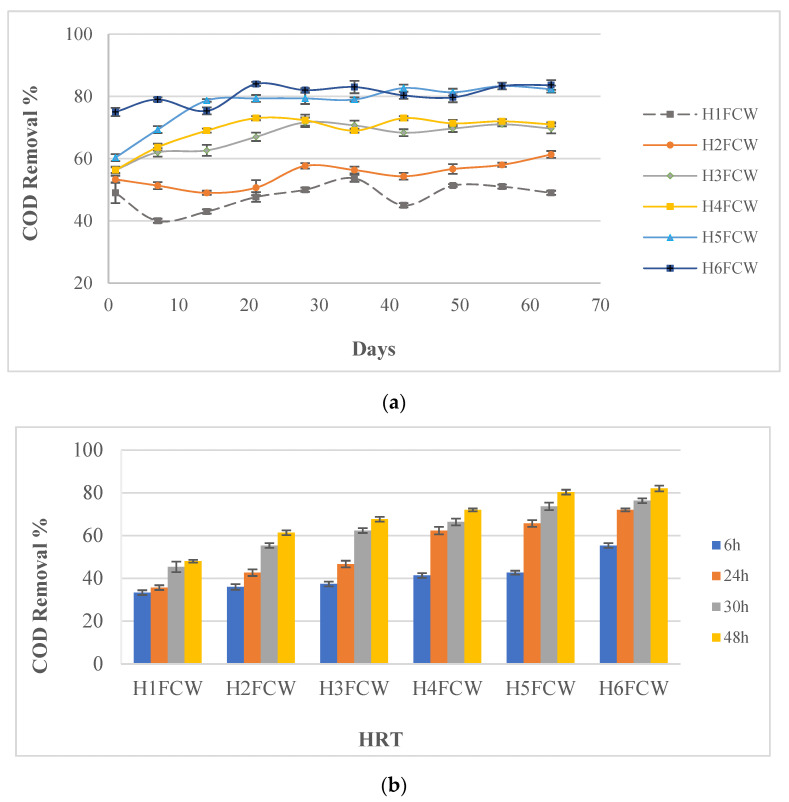
(**a**) COD efficiency removal in different mesocosm horizontal flow constructed wetlands (HFCWs) after 48 h. (**b**) Hydraulic retention time effect on the COD removal.

During the study period, the COD average concentration recorded in the wastewater influent was 474.8 ± 29.48 mg L^−1^. Its removal efficiency in the six constructed wetlands (CWs) after 48 h is shown in Figure 3a. Significant differences (*p* < 0.05) were detected between the six configurations for the COD removal. The highest COD removal was attributed to the units filled with 75% gravel amended with 25% granulated cork (H5FCW and H6FCW), with removal rates of 77.57 ± 5.09% and 80.53 ± 2.66%, respectively. These rates were in harmony with several other publications reporting high COD removal in CWs [50,51,52]. The lowest removal efficiency of 47.97 ± 3.24% was recorded in the unplanted unit filled only with gravel (H1FCW). However, the COD removal efficiency of the unplanted unit filled with gravel amended with cork (H2FCW) was significantly higher than that of H1FCW with a removal rate of 54.87 ± 3.13%. These results indicate that the removal capacity was remarkably promoted upon the utilization of cork as an emerged substrate. Sanjrani [53] stated that the media matrix plays a major role in COD removal efficiency as it can afford a good living condition for the development of microorganisms on its surface area, so they can better achieve different processes such as the adsorption, absorption, and degradation of water pollutants. Indeed, cork has a large specific surface area and a good pore structure, which provides a suitable condition for microbial growth [47]. On the other hand, the COD removal rate in the planted system (H3FCW) exceeded 65%, while it was only 47.97 ± 3.24% in the unplanted unit (H1FCW). Several studies comparing the COD removal efficiency of planted and unplanted systems have revealed that planted CWs outperform unplanted CWs [54,55,56]. In addition, the study carried out by Xu and Cui [57] showed that the substrate interception and adsorption of organic compounds in planted systems were more extensive than those in the unplanted systems_._ The successful COD removal can be attributed to the heterotrophic bacteria using plant interposed dissolved oxygen to promote aerobic oxidation of organic matter [58]. However, the present study showed that H4FCW planted with *Typha* was slightly higher (*p* > 0.05) than H3FCW planted with *Phragmites,* with removal rates of 69.07 ± 3.65% and 66.90 ± 3.94%, respectively. Similarly, Timotewos [59] showed a similar finding with three macrophyte species and *Typha angustifolia* had a slightly better COD removal. Moreover, other studies have reported small differences in the COD removal between ten beds planted with five different macrophytes species_._ These authors showed that the average COD effluent concentration was 26 ± 18 mg L^−1^ in the *Cyperus parirus* CW, 27 mg L^−1^ ± 13 in the *Vetiveria zizanoides* CW, and the lowest value of 25 mg L^−1^ ± 13 was detected in the *Phragmites australis* CW [60]. 

As shown in Figure 3b, the COD removal rate in the system filled with granulated cork (H5FCW) exceeded 60% at 24 h, while it was only 46.67% in H3FCW. An accretion of approximately 38% was observed as the HRT increased from 6 h to 48 h in the H5FCW unit. It was obvious that the HFCW performance depends on the contact period between the system and the wastewater, particularly when HRT was lower than 24 h, especially in the planted systems where the maximum COD removal rates were obtained earlier. For example, the planted unit (H6FCW) with *Typha angustifolia* and filled with gravel amended with cork displayed a COD removal accretion by only 10% upon the HRT increase from 24 h to 48 h. A study conducted by Abed [61] stated that a HRT of 24 h was sufficient for pollution removal from wastewater using the vegetation and media activities. To reach higher COD removal rates (around 91.9%), the HRT must be increased to above 8 days [62]. In contrast, Ballestros [63] showed that increasing the HRT higher than 8 days did not considerably increase the efficiency anymore and would require large-sized CWs. Furthermore, Vymazal [64] showed that excessive retention periods will have adverse effects. In this study, in all the CWs systems, the higher HRT of 2 days reported a higher COD removal and maintained the stability of the treatment efficiency during the experimental period. 

### 3.3. Nitrogen Removal Efficiency

**Figure 4:**
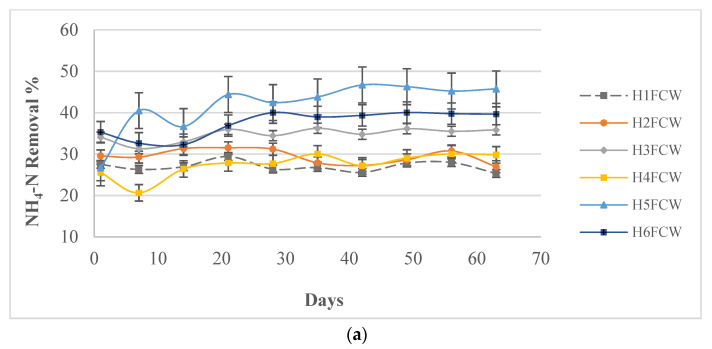

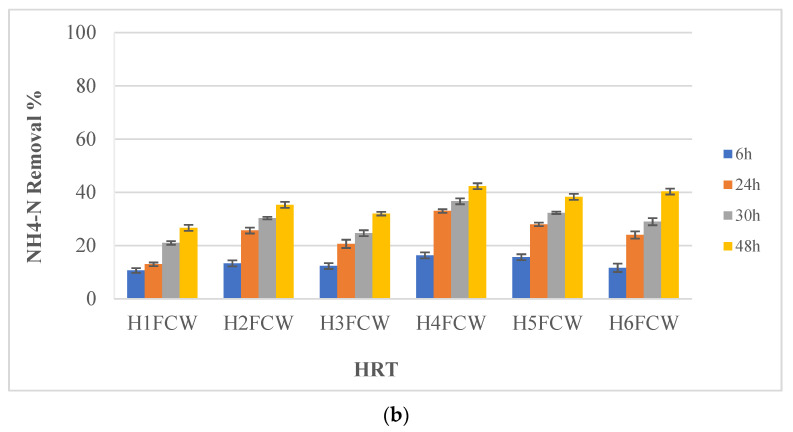
(**a**) Ammonium removal rates in different mesocosm HFCWs after 48 h. (**b**) Hydraulic retention time effect on ammonium removal.

Overall, as for the results of COD removal, the planted units filled with granulated cork contributed to the best enhanced ammonium reduction by 42 ± 4% and 37 ± 3% for H5FCW and H6FCW, respectively. However, the results of units filled only with gravel indicated lower removal efficiency percentages after 48 h (Figure 4a). The lowest removal rate of 27 ± 0.95% attributed to the unit filled only with gravel (H1FCW) was greater than previous studies by Xu [65], reaching 19% for gravel-based CWs. On the other hand, these rates were lower than those reported by Nguyen [66], reaching 85% and using a biochar amendment known for its high nitrogen removal rates. However, biochar presents some disadvantages, principally its lower surface area aside from its loss of activity, which makes it unsuitable for large-scale remediation [37]. The addition of granulated cork significantly boosted the ammonium removal as it effectively improved the porosity within the CWs [67]. Hence, a high rate of oxygen diffusion within the system was increased, entailing an abundant aerobic zone for the growth and reproduction of nitrifying bacteria and achieving an efficient nitrification process. Compared to the COD removal rates, the ammonium removal efficiencies were lower. This particular result was observed in most of the CW systems [66,68].

HRT plays an important role as it affects the contact duration between pollutants and microbes [69]. The removal rates of the different systems continued to increase significantly with the HRT increase (from 6 h until 48 h). The average NH_4_-N removal in the unplanted unit H1FCW filled only with gravel was linearly increased by 10% at 6 h, 13% at 24 h, and 26% at 48 h HRT. On the other hand, the NH_4_-N removal at H2FCW, H3FCW, H4FCW, H5FCW, and H6FCW outlet recorded an average between 32% and 42% at 48 h HRT (Figure 4b). 

Moreover, the ammonium nitrogen removal rate was not stable in all systems. Hence, the ammonium removal required some time to become balanced. In the current study, the ammonium removal in each system increased with the hydraulic retention time. The average removal efficiency of ammonium in the planted units was always higher than the unplanted ones. The results also showed significant differences (*p* < 0.05) between systems in ammonium removal due to the macrophyte species. Carrasco-Acosta [70] and Kraiem [52] reported the multiple effects that macrophytes may have on nutrient removal as it improves the hydraulic conductivity, enhances the biofilm development, and then influences the microbial activity by transferring the oxygen from the atmosphere to the substrate and ensuring enhanced aeration of the bed. Otherwise, the roots can provide adhesion and oxygenation to microbes [71]. *Phragmites australis* CWs provided the lowest concentration and the highest removal rate. This can be attributed to the development of the *Phragmites australis* root system in comparison with *Typha angustifolia,* which increased the oxygenation rate in the media, favoring the activity of ammonia-oxidizing bacteria [72]. Kraiem [52] reported that the macrophyte species had a significant effect on NH_4_-N removal efficiency, which was higher in CWs planted with *Phragmites australis* than in CWs planted with *Typha angustifolia.* Indeed, these authors indicated that the dry weight of the *Phragmites australis* roots was higher than that of *Typha angustifolia.*

**Figure 5:**
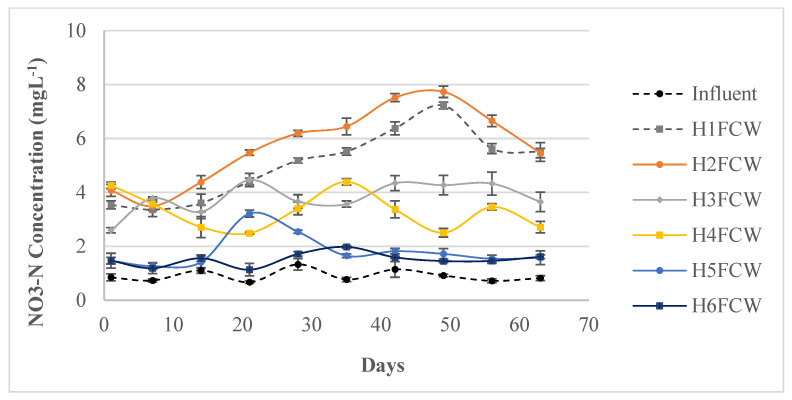
Nitrogen concentration in different mesocosm HFCWs (HRT = 48 h).

In this study, the influent showed an average nitrate nitrogen (NO_3_-N) concentration of 0.95 ± 0.20 mg L^−1^. The HFCW effluents were higher than the influent (Figure 5). The NO_3_-N concentration was significantly lower (*p* < 0.05) in effluents of H6FCW amended with gravel and cork than in H4FCW filled only with gravel. These results may be explained by the effect of the addition of cork, offering a rich carbon source that can significantly induce the full occurrence of the nitrification process. More specifically, it could have played a role as a supplementary carbon source, promoting the reduction of NO_3_- and producing N_2_ by denitrifying bacteria [58].

### 3.4. Investigated Endocrine-Disrupting Chemicals Removal Efficiency

To assess the endocrine-disrupting compound (EDC) removal efficiency, bisphenol A (BPA) and diclofenac (DCF) were selected as representative compounds. Among the efficient CW systems for COD and nitrogen removal, H6FCW planted with *Typha angustifolia* and filled with gravel amended with granulated cork was compared to the unit planted with the same macrophyte species and filled only with gravel (H4FCW) for the media effect evaluation. EDC removal performances of the selected CWs are shown in Table 4. It is noteworthy to mention that several studies found higher removal efficiencies in planted CWs compared to unplanted filters, as the rhizosphere of these aquatic plants host the bacteria involved in pollutant biodegradation [73,74]. Furthermore, *Typha* has been promoted as it shows a strong capability of remediating pharmaceuticals and personal care products (PPCPs) that has been proven, despite the fact that the effectiveness depends on the physical and chemical properties of the contaminants concerned [75]. BPA and DCF were selected for this investigation, as they are frequently detected in wastewaters with influent concentrations detected at 54.47 ± 0.35 μg L^−1^ and 6.35 ± 0.57 μg L^−1^, respectively. The H6FCW filled with gravel amended with cork significantly showed the highest BPA removal rates (*p* < 0.05) of around 90.95 ± 0.37% versus 63.58 ± 0.62% for H4FCW. These results suggest that the following new and valuable insights by using granulated cork as a media substrate could lead to highly effective and stable long-term BPA removal by CWs. The elimination achieved for BPA in this study is in accordance with that reported by Avila [76] using gravel based-CWs under anaerobic conditions (85 and 99%). These authors indicated that BPA removal could be associated with sorption onto organic matter and biodegradation. Toro-Vélez [77] showed BPA removal rates around 73.3 ± 19% in the horizontal subsurface flow CW filled with gravel and cultivated with different macrophyte species *Heliconia psittacorum*, which was higher than H4FCW filled with the same media but remained lower than the rates reached by H6FCW amended with granulated cork. On the other hand, Papaevangelou [78] also investigated the BPA removal efficiency in pilot-scale horizontal subsurface flow CWs filled with gravel and obtained average removals of 49.6% in units planted with *Phragmites australis* and 50% in CWs planted with *Typha latifolia*, which was lower than that found in this study. Furthermore, a study conducted by Carranza-Diaz [79] showed the lowest removal rates in horizontal CWs, around 5–15%.

On the other hand, the DCF removal efficiencies showed similar rates to the BPA results with the highest and most significant removal rates (*p* < 0.05) dedicated to H6FCW with 89.66 ± 0.16% versus 64.52 ± 0.38%. Ilyas and van Hullebusch [80] showed that the DCF removal efficiency in HFCWs filled with gravel amended with sand was around 39 ± 24%, which was lower than that found in this study. Zhang [81] investigated the ability of tropical horizontal subsurface CWs filled with gravel and planted with *Typha angustifolia* to remove the pharmaceutical compounds. DCF removal efficiency in the planted units ranged from 47.5 to 55.4% compared to that of the unplanted units that ranged between 41.1 and 46.7%. Furthermore, Zhang [82] illustrated that photodegradation, biodegradation, and plant uptake were the fundamental removal mechanisms for EDC removal in CWs_._ Thus, DCF was classified by Mathon [83] as a fast-photodegradable compound. Granulated cork is considered as an emerged substrate utilized in CWs. It has been shown to be capable of significantly retaining even some organic pollutants such as some pesticides [84]. Furthermore, granulated cork has been used to remove ibuprofen, carbamazepine, and clofibric acid, showing a good sorption ability [85]. However, the cork capacity of BPA and DCF removal in CWs have still not been widely investigated and highlighted. Therefore, this part could be regarded as the main novelty of this work in comparison with previous studies.

## 4. Conclusions

In the present work, wastewater treatment by six mesocosm horizontal flow constructed wetlands (HFCWs) filled only with gravel or gravel amended with granulated cork was investigated. The results revealed that the planted HFCWs outperformed the unplanted ones and both macrophyte species *Phragmites australis* and *Typha angustifolia* showed steeper efficiencies. On the other hand, the systems amended with granulated cork achieved a higher COD after 24 h. However, ammonium nitrogen and nitrate nitrogen needed more contact time to be removed. Among the efficient CW systems for COD and nitrogen removal, two systems planted with *Typha* chosen as the cattail species were tested and confirmed for its potential EDC removal efficiency. Two systems were compared, from one part (H6FCW) filled with gravel amended with granulated cork that showed higher COD and nitrogen removal and from the second part (H4FCW) filled only with gravel, in order to investigate the media effect on two representative endocrine-disrupting compounds (EDCs), namely bisphenol A (BPA) and diclofenac (DCF). The results showed a high removal efficiency for both pollutants with removal rates of 90.95 ± 0.37% and 89.66 ± 0.16%, for BPA and DCF, respectively. In summary, cork can be used as a novel adsorbent of micropollutants, as it presents different advantages unlike other adsorbents, with no pretreatment required. Further tests can be widened to evaluate the optimal dose of amended granulated cork in a real full-scale.

## Figures and Tables

**Table 1:** Influent characteristics and water quality standards for wastewater reuse in agriculture. [42,43,44].

Influent		Standards for Agricultural Reuse			
		Tunisian Limits (NT106.03)	European Union Limits	WHO (2006) Wastewater Quality for Agriculture	EPA (USA)
pH	7.8 ± 0.3	6.5–8.5	6–9.5	5.8–8.5	6–9
EC (µS /cm)	5600 ± 215	7000	n.r	4500	n.r
TSS (mg L^−1^)	269 ± 22.7	≤30	A: ≤10 B: ≤35 C: ≤35 D: ≤35	Unrestricted <50 Restricted 50–100	≤30
COD (mg L^−1^)	474.8 ± 29.48	≤90	n.r	n.r	n.r
BOD_5_ (mg L^−1^)	230 ± 20	≤30	A: ≤10 B: ≤25 C: ≤25 D: ≤25	n.r	For food crops: ≤10 Industrial crops: ≤30
NH_4_-N (mg L^−1^)	83.57 ± 2.95	n.r	n.r	n.r	n.r
NO_3_-N (mg L^−1^)	0.95 ± 0.20	n.r	n.r	n.r	n.r

Results of the influent characteristics are presented as the means ± standard deviations. Abbreviations: n.r: not recorded—no data.

**Table 2:** Main configurations of the horizontal flow constructed wetlands (HFCWs).

Unit	Substrate	Plant	Volume (L)
H1FCW	100% Gravel	Unplanted	21
H2FCW	75% Gravel + 25% Granulated Cork	Unplanted	21
H3FCW	100% Gravel	*Phragmites australis*	21
H4FCW	100% Gravel	*Typha angustifolia*	21
H5FCW	75% Gravel + 25% Granulated Cork	*Phragmites australis*	21
H6FCW	75% Gravel + 25% Granulated Cork	*Typha angustifolia*	21

**Table 3:** Chemical formula/structure, molecular weight, molecular mass of the derivative compounds, quantifier ions (*m/z*), and correlation coefficient (R^2^) of the target EDCs.

Compounds	Chemical Formula	Chemical Structure	Mw (g mol^−1^)	Mw -TMS	Ions (*m/z*)	R^2^
Bisphenol A (BPA)	C_15_H_16_O_2_	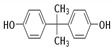	228.29	372	357 358	0.990
Diclofenac (DCF)	C_14_H_10_Cl_2_NO_2_Na	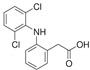	318.1	367	214 242 277	0.996

**Table 4:** Concentrations of the target EDCs recorded in the influent and effluent and their removal efficiency in the selected constructed wetlands.

Target EDCs	Concentration Average			EDCs Removal Rates %	
	Spiked Influent (µg L^−1^)	H4FCW Effluent (µg L^−1^)	H6FCW Effluent (µg L^−1^)	H4FCW	H6FCW
Bisphenol A (BPA)	74.47 ± 0.35	27.12 ± 0.43	6.74 ± 0.62	63.58 ± 0.62	90.95 ± 0.37
Diclofenac (DCF)	26.35 ± 0.57	9.35 ± 0.48	2.72 ± 0.53	64.52 ± 0.38	89.66 ± 0.16

## Data Availability

The authors declare that all data supporting the findings of this study are available within the article and its supplementary information files.
